# Editorial: Tumour microenvironment in cancer research and drug discovery

**DOI:** 10.3389/fphar.2024.1403176

**Published:** 2024-05-03

**Authors:** Nur Akmarina B. M. Said, Syed Mahmood, Kenneth K. W. To

**Affiliations:** ^1^ Department of Pharmaceutical Life Sciences, Faculty of Pharmacy, University of Malaya, Kuala Lumpur, Malaysia; ^2^ Department of Pharmaceutical Technology, Faculty of Pharmacy, University of Malaya, Kuala Lumpur, Malaysia; ^3^ School of Pharmacy, Faculty of Medicine, The Chinese University of Hong Kong, Hong Kong, Hong Kong SAR, China

**Keywords:** cancer, tumor microenvironment (TME), drug repurposing, immunotherapy, immune cells

It has been long accepted that tumours do not exist or function in silos; indeed tumour progression is the orchestrated consequence of paracrine networking between tumours and their surrounding factors. Considering the tumour microenvironment (TME) in cancer research has profoundly shifted the paradigms in molecular investigation and translational drug discovery.

The TME components, including immune-inflammatory cells, cancer-associated fibroblasts, and endothelial and adipose cells, are correlated with cancer signatures, which carry diagnostic, therapeutic, and predictive values. There has been evidence to suggest changes in these microenvironmental cell types play an important role in even from somatic clonal expansions (Munugula et al.) and potentially the initial stages of cancer progression in normal tissues. In this Research Topic, we highlighted the significant impact of the TME component on cancer research, with a special focus on immune cells and drug repurposing targeting this component.

Immune cells surrounding tumours can be regulated by the metabolic microenvironment, which further reshapes the immune landscape. A myriad of key molecular regulators involved in lipid metabolism and glucose homeostasis have been implicated in reshaping the TME as well as assisting immune infiltration towards tumour progression and treatment resistance. In this edition, proliferator-activated receptor gamma (PPARγ) (Ran et al.) is comprehensively analysed.

Cancer metabolism takes place from the level of cellular metabolism down to nucleotide/amino acid metabolism. In glioma with active tryptophan catabolism and less tryptophan in the tumour, their high tryptophan metabolism-related genes signature (TrMRS) (Zhang et al.) was correlated with more immune cell infiltration and a “hot” immunological phenotype. Interestingly, this immuno-metabolic correlation holds the predictive value towards patients’ response to immune checkpoint inhibitors.

Indeed, the involvement of immune cells in cancer progression has guided many treatment choices. Intensive lymphocyte infiltration has been shown to occur in higher-grade or recurrent gastric tumours (Zheng et al.). Immune cell infiltration has also been linked to m7G RNA methylation (Chen et al.) in renal clear cell carcinoma, with the proposed m7G score being used to predict the cancer prognosis. In glioma too, the necroptosis index (NI) (Ma et al.) has been shown to influence sensitivity to immunotherapy, particularly anti-PD1 therapy. The prediction of the immunotherapy response has become the center of attention in studies evolving around TME. While this is not surprising given immune cells are the predominant gatekeeper cells that are often compromised in cancer, questions remain about whether the myriad of predictive data could be supported with strong mechanistic studies, both *in vitro* and *in vivo*.

Drug repurposing in targeting the TME is another Research Topic of interest in this area. It is very interesting to see how non-cancer-targeting substances/drugs could influence the immune landscape to the extent that they affect the response towards immunotherapy. This category includes aspirin and statins, which have been demonstrated to target the metabolic and immunomodulatory landscape of TME in preclinical models (Yu et al., 2022; Jing et al., 2023). It is more intriguing to see that most of the drugs being researched are ‘accessory’ drugs that are often being used by cancer patients in one way or another.

In this special edition, an antidepressant is shown to not only alleviate the depressant condition of cancer patients but also strengthen antitumour immunity. When using the antidepressant drug ansofaxine hydrochloride (Jing et al.), it is demonstrated this is achieved by the exhaustion of CD8+T, which also enhances the antitumour effects of anti-TNFR2.

Another drug—propofol anaesthesia usually administered during surgical resection of solid tumours—has been clinically shown to contribute to a better postoperative outcome in some malignant tumour surgeries, and it participates in reshaping the TME. Specifically, anti-angiogenesis was observed along with regulation of immunity, reduction of inflammation, and remodelling of the extracellular matrix. In this special edition, propofol (Zhou et al.) is extensively and mechanistically reviewed to provide a better understanding of its role in modulating TME with a theoretical guide for the selection of anaesthetics used in malignant tumour surgery.

A combination treatment using cordycepin (the vital bioactive compound of *Cordyseps* (Feng et al.) that has been used for its therapeutic potential) and the anti-CD47 antibody has been demonstrated to significantly enhance tumour suppression. This is in parallel with the increase in the proportion of M1 macrophages and the decrease in the M2 macrophages proportion. M1 and M2 are the two distinctive phenotypes of tumour-associated macrophages (TAMs), where M1 is known to inhibit tumour progression by inducing inflammatory responses and M2 supports tumour progression by suppressing immune responses.

Despite the concerted effort in the development of drugs targeting TME, such as BLZ945 (a colony-stimulating factor-1 receptor inhibitor) (Wei et al., 2020), there is a gap in the market for clinically satisfactory drugs that target this intricate factor. Among the highly researched is Focal adhesion kinase (FAK) (Hu et al.)—a non-receptor tyrosine kinase reviewed in this special edition. Numerous FAK inhibitors, including IN10018, defactinib, GSK2256098, conteltinib, and APG-2449, have demonstrated positive antitumour effects in preclinical studies and are undergoing clinical trials for their tumour scaffold function in sustaining TME.

siRNA and miRNA nanotherapeutics targeting TME molecular components is another tool being employed owing to their powerful gene-silencing properties, although formulation for effective delivery remains a challenge. In this context, it has been demonstrated that T7 peptide-decorated exosomes were able to package and protect cholesterol-modified Cy3-siYY1 (siRNA for Yin Yang transcription factor) (Liu et al.) to be released in the cytoplasmic reductive environment that is often observed in the tumourigenesis of glioblastoma.

The translational impact of the manipulation of the TME components is yet to be ascertained, but the landscape shows promise. The combination of a new treatment targeting TME with standard chemotherapy/immunotherapy also consistently demonstrates synergistic effects, and it will be very interesting to see this extended to targeting cell types other than immune cells within the TME in this regard.
